# Downregulation of FIP200 Induces Apoptosis of Glioblastoma Cells and Microvascular Endothelial Cells by Enhancing Pyk2 Activity

**DOI:** 10.1371/journal.pone.0019629

**Published:** 2011-05-13

**Authors:** Dongyan Wang, Mitchell A. Olman, Jerry Stewart, Russell Tipps, Ping Huang, Paul W. Sanders, Eric Toline, Richard A. Prayson, Jeongwu Lee, Robert J.Weil, Cheryl A. Palmer, G. Yancey Gillespie, Wei Michael Liu, Russell O. Pieper, Jun-Lin Guan, Candece L. Gladson

**Affiliations:** 1 Division of Neuropathology, Department of Pathology, University of Alabama at Birmingham, Birmingham, Alabama, United States of America; 2 Division of Pulmonary/Critical Care, Department of Medicine, University of Alabama at Birmingham, Birmingham, Alabama, United States of America; 3 Department of Nephrology, University of Alabama at Birmingham, Birmingham, Alabama, United States of America; 4 Neurosurgery Division, Department of Surgery, University of Alabama at Birmingham, Birmingham, Alabama, United States of America; 5 Department of Cancer Biology, Cleveland Clinic, Cleveland, Ohio, United States of America; 6 Department of Stem Cell and Regenerative Medicine, Cleveland Clinic, Cleveland, Ohio, United States of America; 7 Department of Anatomic Pathology, Cleveland Clinic, Cleveland, Ohio, United States of America; 8 Brain Tumor and Neuro-Oncology Center, Cleveland Clinic, Cleveland, Ohio, United States of America; 9 Department of Neurosurgery, University of California San Francisco, San Francisco, California, United States of America; 10 Division of Molecular Medicine and Genetics, Cell and Developmental Biology, Department of Internal Medicine, University of Michigan, Ann Arbor, Michigan, United States of America; Yale Medical School, United States of America

## Abstract

The expression of focal adhesion kinase family interacting protein of 200-kDa (FIP200) in normal brain is limited to some neurons and glial cells. On immunohistochemical analysis of biopsies of glioblastoma tumors, we detected FIP200 in the tumor cells, tumor-associated endothelial cells, and occasional glial cells. Human glioblastoma tumor cell lines and immortalized human astrocytes cultured in complete media also expressed FIP200 as did primary human brain microvessel endothelial cells (MvEC), which proliferate in culture and resemble reactive endothelial cells. Downregulation of endogenous expression of FIP200 using small interfering RNA resulted in induction of apoptosis in the human glioblastoma tumor cells, immortalized human astrocytes, and primary human brain MvEC. It has been shown by other investigators using cells from other tissues that FIP200 can interact directly with, and inhibit, proline-rich tyrosine kinase 2 (Pyk2) and focal adhesion kinase (FAK). In the human glioblastoma tumor cells, immortalized human astrocytes, and primary human brain MvEC, we found that downregulation of FIP200 increased the activity of Pyk2 without increasing its expression, but did not affect the activity or expression of FAK. Coimmunoprecipitation and colocalization studies indicated that the endogenous FIP200 was largely associated with Pyk2, rather than FAK, in the glioblastoma tumor cells and brain MvEC. Moreover, the pro-apoptotic effect of FIP200 downregulation was inhibited significantly by a TAT-Pyk2-fusion protein containing the Pyk2 autophosphorylation site in these cells. In summary, downregulation of endogenous FIP200 protein in glioblastoma tumor cells, astrocytes, and brain MvECs promotes apoptosis, most likely due to the removal of a direct interaction of FIP200 with Pyk2 that inhibits Pyk2 activation, suggesting that FIP200 expression may be required for the survival of all three cell types found in glioblastoma tumors.

## Introduction

The focal adhesion kinase family interacting protein of 200-kDa (FIP200), which is also known as retinoblastoma coiled coil protein 1 (Rb1CC1), can interact with and regulate the activity of several different proteins that are involved in the signaling of proliferation, apoptosis, autophagy, and cell cycle progression [1]–[4] and is therefore particularly well-positioned to regulate cell survival. The FIP200-nullizygous state is embryonic lethal in mice and E14.5–E15.5 day embryos show histologic evidence of heart failure and liver degeneration that is associated with high levels of apoptosis in the heart and liver tissue [5]. The *FIP200* gene is highly conserved and is expressed in a wide variety of tissues and cell lines [2], [6]; however, its effects appear to be highly dependent on the cell type and the experimental conditions [3].

Several different regulatory mechanisms have been identified that may affect FIP200 activity, including its level of expression [7], localization (cytoplasmic *vs.* nuclear) [1], [3], [8]–[10], and its interaction with other proteins [3], [4], [10]. FIP200 can interact directly with the kinase domains of both focal adhesion kinase (FAK) and proline-rich tyrosine kinase 2 (Pyk2, also known as related adhesion focal tyrosine kinase, calcium-dependent tyrosine kinase, and focal adhesion kinase-2). Overexpression of FIP200 inhibits the kinase activity and the kinase-associated cellular functions of these proteins [1], [2]. Both Pyk2 and FAK regulate cell proliferation, apoptosis, and the cell cycle; in general, Pyk2 acts to downregulate cell survival whereas FAK acts to promote it [11]–[17]. The forced overexpression of Pyk2 induces apoptosis, with the N-terminal and kinase domains of Pyk2 being necessary for this effect [11], [18]. Some apoptosis signals [12], [19] activate Pyk2 including TNFα [19]. Clues to the associated signaling pathways are provided by the findings that methylmethane sulfonate induction of apoptosis requires Pyk2 activation of JNK [20] and expression of the transcription factor Smad4 can increase Pyk2 expression with a subsequent promotion of apoptosis in MDA-MB468 cells [21].

Evidence that Pyk2 can modulate FAK activity has been generated through analysis of FAK-mediated promotion of cell cycle progression. FAK can promote cell cycle progression through activation of ERK and induction of the transcription factor Kruppel-like factor 8 (KLF8), which activates transcription of cyclin D1, as well as through inhibition of p27(Kip1) expression in glioblastoma cells [13]–[15], [17]. The C-terminal domain of Pyk2 is necessary for its inhibition of the cell cycle [13] and expression of Pyk2 in cells with a low level of endogenous Pyk2 inhibits cell cycle progression through the differential activation of JNK and ERK [13]. Notably, Pyk2 also can inhibit cell cycle progression by competing with endogenous FAK for binding partners. Specifically, Pyk2 can effectively compete with FAK for binding to Src and/or Fyn and thereby inhibit ERK activation [13].

The analysis of the potential role of FIP200 in tumor development has focused, for the most part, on breast cancer [7], [22], [23], and there has been one report of the effects of transient downregulation of FIP200 in breast tumor cells [7]. We investigated whether endogenous FIP200 protein regulates the proliferation of cells found in glioblastoma tumors and whether it does so by regulating Pyk2 and/or FAK activity. We and others have shown previously that Pyk2 and FAK can regulate cell proliferation and survival in glioblastoma cells, with overexpression of Pyk2 decreasing and overexpression of FAK enhancing cell proliferation [17], [24], [25]. In the current studies, we found that FIP200 is expressed in tumor cells and occasional glial cells in glioblastoma tumor biopsies. It also was present in the endothelial cells in the tumor-associated vessels, although it is not detectable in the endothelial cells in the vessels of normal brain. siRNA downregulation of endogenous FIP200 protein inhibited cell proliferation and induced apoptosis in U-87MG human glioblastoma cells, immortalized human astrocytes, and human brain microvessel endothelial cells (MvECs) propagated in complete media, which are reactive and commonly used as a surrogate model of tumor-associated MvECs. FIP200 downregulation increased Pyk2 activity, without affecting FAK activity. The pro-apoptotic effect was blocked by a fusion protein containing the Pyk2 autophosphorylation site in the glioblastoma cells and the MvECs. This preferential effect of the FIP200 downregulation on Pyk2 activity may be explained by the greater association of FIP200 with Pyk2, rather than FAK. Our data suggest that FIP200 may be necessary for the survival of multiple cell types found in glioblastoma tumors, and that FIP200 inhibition of Pyk2 activity may play a prominent role in this promotion of survival.

## Results

### FIP200 Protein is Expressed in Tumor Cells, Glial Cells and the Endothelial Cells of Tumor-Associated Vessels in Glioblastoma Biopsies

**Figure 1:**
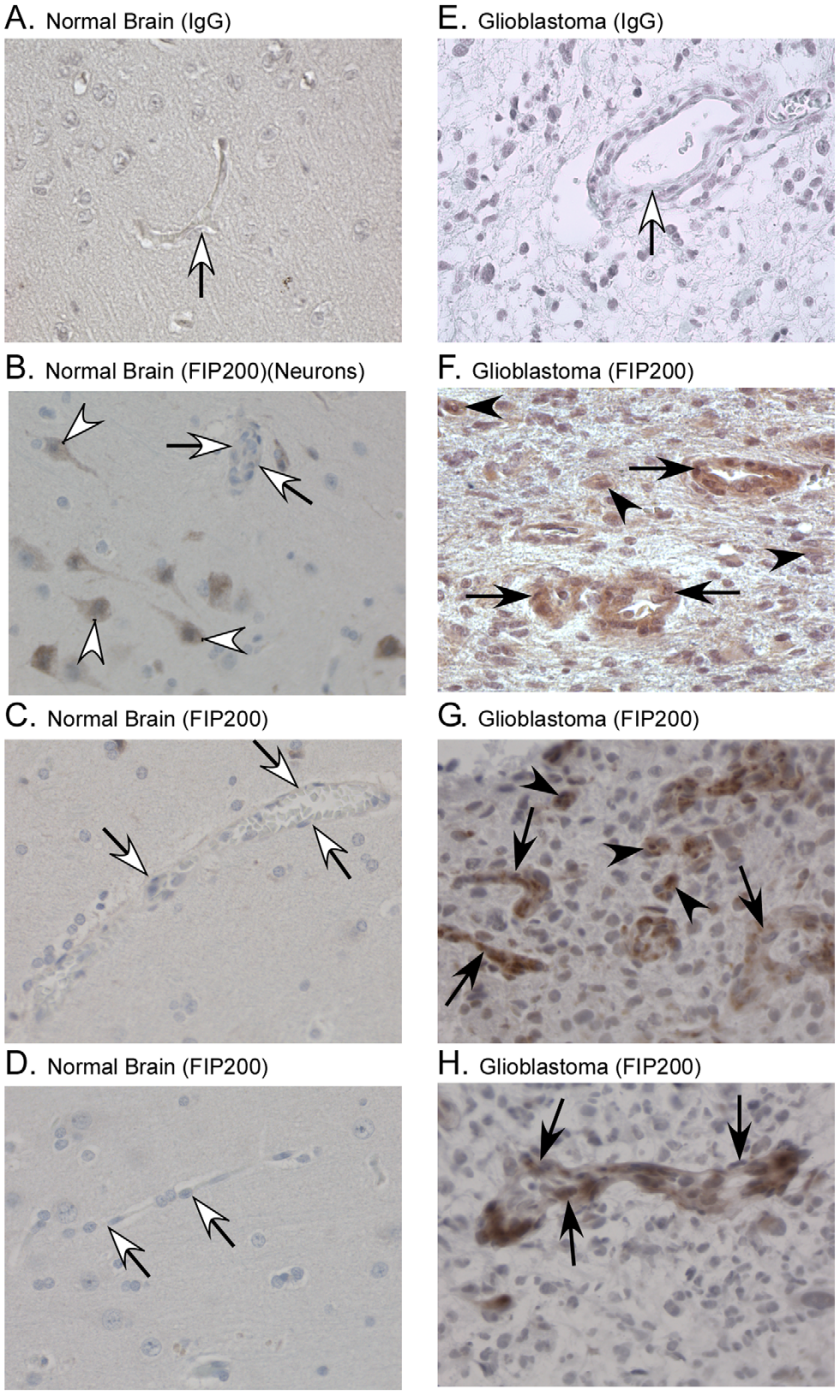
FIP200 is expressed in tumor-associated endothelial cells as well as tumor cells in glioblastoma tumor biopsies. Formalin-fixed and paraffin-embedded sections of surgical biopsies of normal brain (n = 20) and glioblastomas (n = 21), as well as frozen sections of glioblastoma (n = 15) were subjected to immunohistochemical analysis using diaminobenzidene as the substrate and counterstaining with hematoxylin. The sections were reacted with a commercial rabbit anti-FIP200 (0.2 microgram/ml) or rabbit IgG (0.2 microgram/ml) for 24 h (4°C). A–D, representative photomicrographs of sections of normal brain reacted with control rabbit IgG (A) or rabbit anti-FIP200 (B–D). Open arrowheads, neurons; open arrows, unstained endothelial cells. Magnification = 400×. E–H, representative photomicrographs of glioblastoma tumor sections reacted with 0.2 microgram/ml control rabbit IgG (E) or rabbit anti-FIP200. F–G, representative micrographs of sections of glioblastoma tumors in which tumor cells expressed FIP200 (28 of 36). Filled arrow-head, tumor cell expressing FIP200; filled arrows, endothelial cells expressing FIP200. Sections were viewed and photographed using a Leica DM4000 microscope. Magnification, 400×.

Immunohistochemical analysis of the expression of FIP200 in formalin-fixed, paraffin-embedded biopsies was carried out using a rabbit anti-FIP200 antibody used previously to identify FIP200 expression in neurons in the brain [10]. Representative photomicrographs are shown in Figure 1. In all of the 20 normal brain biopsies, FIP200 was detected in the cytoplasm of a population of neurons (Figure 1B) and in the cytoplasm of occasional glial cells (data not shown); with the FIP200 being largely localized to the cortex. The detection of FIP200 in a population of neurons in the normal brain cortex is consistent with its reported role in maintaining normal homeostasis of neurons [10]. In the tumor biopsies from 14 of 21 patients with glioblastoma, FIP200 was detectable in the tumor cells. As can be seen in the representative photomicrographs shown in Figure 1, panel F, the levels of expression varied considerably among the tumor cells in any one glioblastoma biopsy as well as among the biopsies from different patients. FIP200 also was detectable in an occasional glial cell in 19 of the 21 glioblastoma biopsy samples (data not shown). In the tumor cells in the glioblastoma biopsies, the FIP200 staining usually was located in the cytoplasm but occasionally was seen in the nucleus, which is consistent with the staining patterns described for FIP200 expression in breast cancer biopsies [26]. In the glial cells it was localized in the cytoplasm. Similar results were obtained on immunohistochemical analysis of 15 additional frozen biopsies (Fig. 1G). FIP200 staining of tumor cells was observed in 14 of these biopsies, and in an occasional glial cell in all of them. The reactivity of the rabbit antibody with FIP200 in the brain biopsies was confirmed by immunoblotting detergent lysates of 17 glioblastoma and five anaplastic astrocytoma, as well as nine normal brain cortex or white matter biopsies (Figure S1 and data not shown). Immunoblotting of U-87MG and SNB19 human glioblastoma cells and 827 and 905 human glioblastoma stem cells propagated as neurospheres with the rabbit antibody showed that these cell lines express FIP200 (Figure S2A).

On examining the gliobastoma biopsies, we observed apparent staining of FIP200 in the endothelial cells of the tumor-associated blood vessels although such staining had not been noted on examination of the normal brain samples. We therefore assessed FIP200 staining in the vessels using a semi-quantitative immunohistochemical approach. The pathological identification of the vessels was confirmed by staining of the von Willebrand factor (vWf) in the endothelial cells in the adjacent sections and only small and medium sized vessels were counted in case the FIP200 staining reflected differences in the size of the vessels. The tumor-associated vessels exhibited the histologic abnormalities described previously [27], *i.e.*, dilated, tortuous, and with reduced pericyte coverage. FIP200 was not detectable in the endothelial cells in the small and medium-sized vessels in 19 of the 20 normal brain biopsies (Figures 1B–D). (In the one biopsy in which staining of the endothelial cells in the vessels was observed, approximately 5% of the blood vessels contained endothelial cells that stained weakly for FIP200.) In marked contrast, FIP200 was detectable in the endothelial cells of the tumor-associated vessels in 16 of the 21 formalin-fixed, paraffin-embedded glioblastoma biopsies and in 14 of the 15 additional frozen biopsies (Fig. 1F–H). (In the five formalin-fixed, paraffin-embedded biopsies in which the endothelial cells in the tumor-associated vessels did not stain for FIP200, the tumor cells also were negative for FIP200 and in the one frozen biopsy that was negative for staining of the endothelial cells, only 10% of the tumor cells were positive for staining of FIP200.) This unexpected difference in expression of FIP200 in the endothelial cells in the glioblastoma tumors and normal brain was significant (p value<0.0001, Fisher exact test). Immunoblot analysis of cultured primary normal human brain endothelial cells propagated in complete media, which are commonly used as a surrogate model for the endothelial cells in brain tumor-associated microvessels, indicated that the rabbit antibody was reacting with FIP200 in these cells (Figure S2B). In the endothelial cells in the tumor-associated vessels in the glioblastoma tumor biopsies, the FIP200 staining was cytoplasmic.

### FIP200 Downregulation Inhibits Proliferation and Induces Apoptosis of Glioblastoma Cells, Immortalized Astrocytes, and Cultured Primary Human Brain MvEC

Two duplex oligonucleotides (FIP200 siRNA #1 and #2) were used to determine the effects of downregulation of endogenous FIP200 protein on proliferation and apoptosis in the three cell types found in glioblastomas. The U-87MG cell line was selected for these studies as it expressed intermediate levels of FIP200 as compared to the other cell lines tested (Figure S2A), immortalized human astrocytes (E6/E7 cells) were used as a model for the tumor-associated glial cells [28], and cultured human brain microvessel endothelial cells were used as a model for the endothelial cells in the tumor-associated vessels [29], [30]. All experiments were carried out using cells propagated in complete media. Transfection of all three cell types with either of the two duplex oligonucleotides used in the experiments effectively downregulated FIP200 protein as assessed by densitometric analysis of immunoblots (Table S1). Controls included a random scrambled siRNA or siFIP200 #1 and #2 duplex oligonucleotides with one or two base pair mutations (mutant siFIP200).

**Figure 2:**
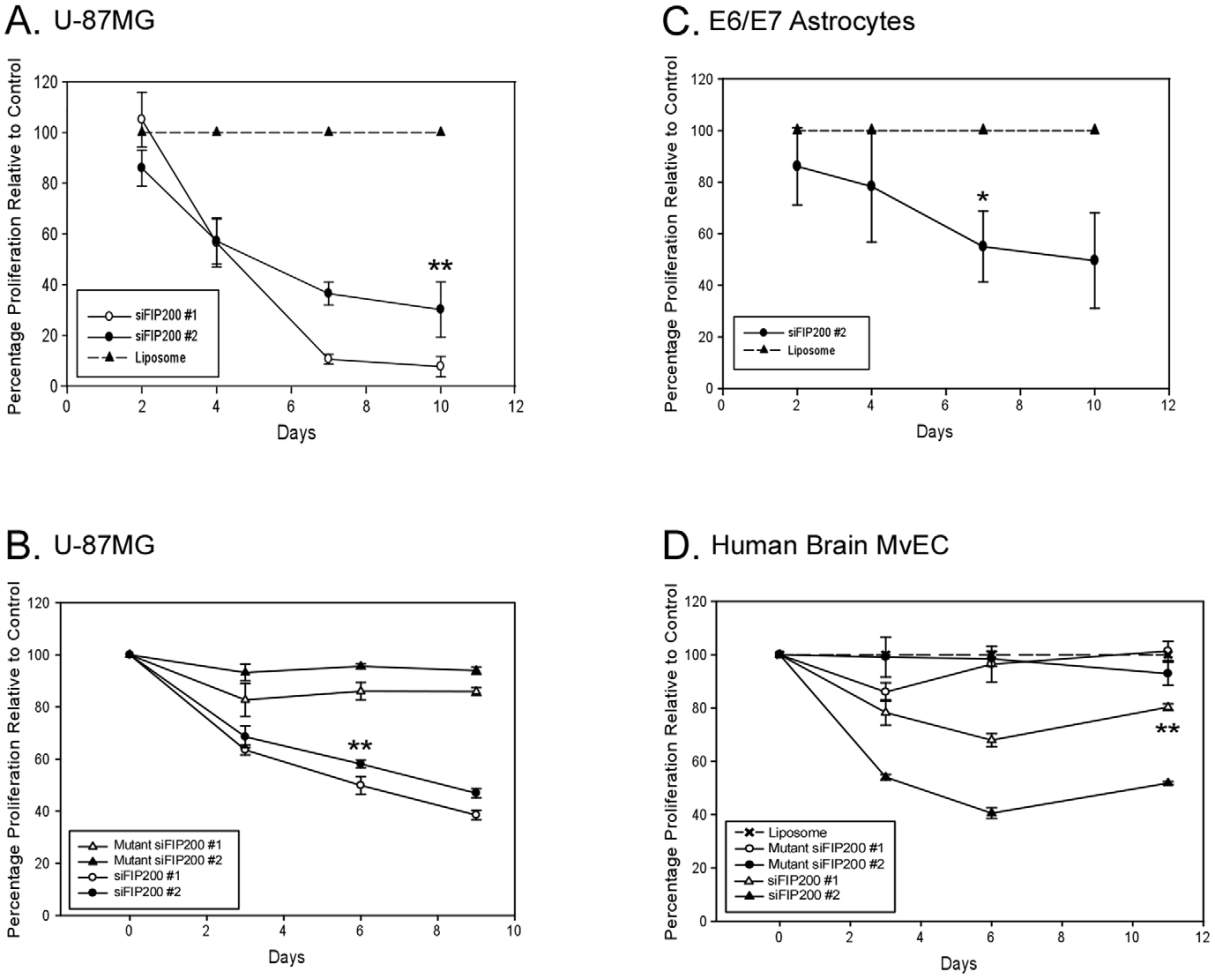
Cell proliferation is inhibited in glioblastoma cells, immortalized astrocytes, and primary human brain MvECs on downregulation of FIP200. U-87MG human glioblastoma cells (A & B) and E6/E7 immortalized human astrocytes (C) were plated in complete media (1×10^4^ cells/well) in replicas of three or four (20 h) then treated with siFIP200 #1 or siFIP200 #2, or with non-silencing siRNA (denoted as liposome) or with mutant siFIP200 #1 or mutant siFIP200 #2 for the indicated time periods with the cell media and siRNA being replaced every three to four days. The cells were harvested and adherent cells counted. (D) Primary human brain MvEC, which are a surrogate model of tumor-associated MvECs and express FIP200, were plated in complete media (10% FBS, 10 ng/ml VEGF, 5 ng/ml bFGF) and treated with siFIP200 #1 or #2 or with mutant siFIP200 #1 or #2 and the adherent cells counted. The means ± S.E.M were calculated and the data are plotted as the percentage of proliferation (cell number) relative to control cells. The experiment was repeated 2× and representative results are shown. (* probability = 0.03; ** probability ≤0.01 as compared to the control at that day; unpaired t test.)

Treatment of the U-87MG cells with either siFIP200 #1 or siFIP200 #2 resulted in significant inhibition of proliferation as compared to treatment with the random scrambled siRNA (Figure 2A) or the mutant siFIP200 #1 and #2 RNAs (Figure 2B). There also was significant inhibition of proliferation when the cells were plated at a higher confluency prior to transfection with siFIP200 #1 or #2 for 7 days (data not shown). Downregulation of FIP200 by treatment of the immortalized human astrocytes (E6/E7 cells) (Figure 2C) and the cultured primary normal human brain endothelial cells (Figure 2D) also resulted in a significant reduction in proliferation.

**Figure 3:**
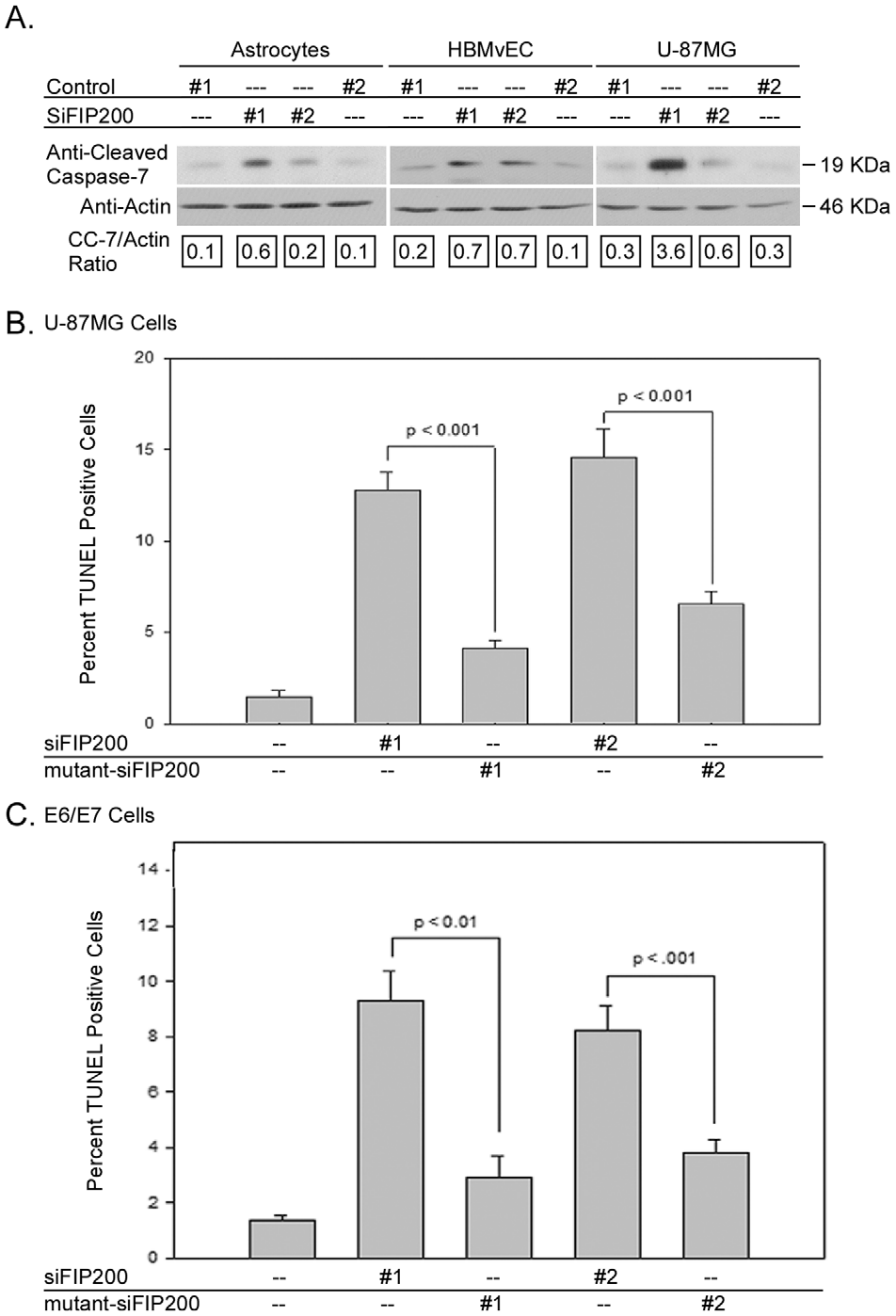
Apoptotic cell death is induced in glioblastoma cells, astrocytes, and primary human brain MvECs on downregulation of FIP200. A, Human astrocytes, U-87MG glioblastoma cells and human brain MvEC (HBMvEC) were plated in complete media (20 h) and then treated with FIP200 siRNA, a non-silencing control siRNA (control #1), or mutant siFIP200 (control #2) for 96 h. Whole cell lysates were resolved on disulfide-reduced 12% SDS-PAGE and blotted with the indicated antibodies as described in the Materials and Methods. The experiment was repeated 2× with the three different cell types and a representative experiment is shown. B & C, U-87MG and E6/E7 E6/E7 immortalized astrocytes were plated in replicas of two, treated with siFIP200 or mutant siFIP200 as described for Panel A for 96 h and then subjected to the TUNEL assay. TUNEL-positive cells were counted in 10 fields per well in a blinded manner and the data analyzed and presented as the mean ± S.E.M. Highly similar results were seen with HBMvEC plated and treated as above with siFIP200 and then subjected to TUNEL assay at 96 h. The experiment was repeated 2× and a representative result is shown.

Transfection of all three cell types with either siFIP200 #1 or #2 for 96 h induced caspase-7 cleavage (Figure 3A) with higher levels of caspase-7 cleavage occurring at day 7 (data not shown). Increased caspase-7 cleavage was observed in the primary and the immortalized human astrocytes on siFIP200 #1 or #2 treatment for 96 h. Similarly, transfection of all three cell types with siFIP200#1 or #2 for 96 h resulted in significantly higher numbers of TUNEL-positive cells than transfection with the mutant siFIP200 #1 or #2 RNAs (Figures 3B & 3C, and data not shown).

### FIP200 Downregulation Increases Pyk2 Activity, but not FAK activity, in Glioblastoma Cells, Immortalized Astrocytes, and Primary Human Brain MvEC

**Figure 4:**
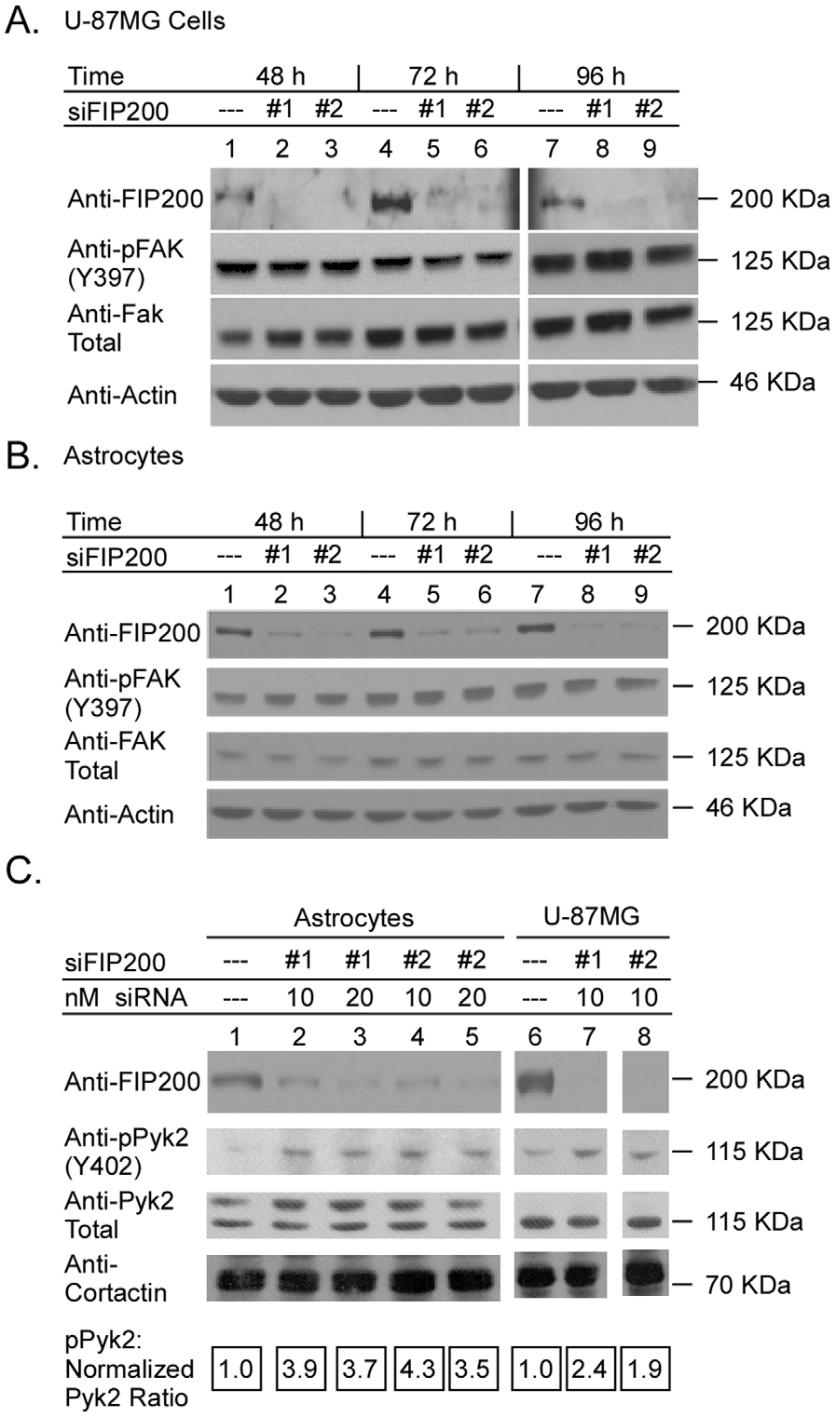
Downregulation of FIP200 protein with siRNA enhances Pyk2 activity without affecting FAK activity in gliobastoma cells and E6/E7 immortalized astrocytes. A, B, U-87MG glioblastoma cells and immortalized human astrocytes were plated in complete media (10% FBS), treated with 20 nM FIP200 siRNA (#1, or #2) or control non-silencing siRNA for the indicated times, detergent lysed, equivalent amount of lysate resolved on disulfide-reduced 6% SDS-PAGE, and then blotted with the indicated antibodies. C, U-87MG glioblastoma cells and immortalized human astrocytes were plated in complete media (10% FBS), and treated with different concentrations of FIP200 siRNA (#1, or #2) or control non-silencing siRNA for 53 h, detergent lysed, equivalent amount of lysate resolved on disulfide-reduced 6% SDS-PAGE, and then blotted with the indicated antibodies. In Panel C all lanes are from the same gel and the densitometric ratio of pPyk2/normalized Pyk2 was set at 1.0 for the cells treated with control non-silencing siRNA, and the ratio for the cells treated with siFIP200 expressed relative to the ratio for the cells treated with control non-silencing siRNA. The experiments were repeated 2× and representative blots are shown.

**Figure 5:**
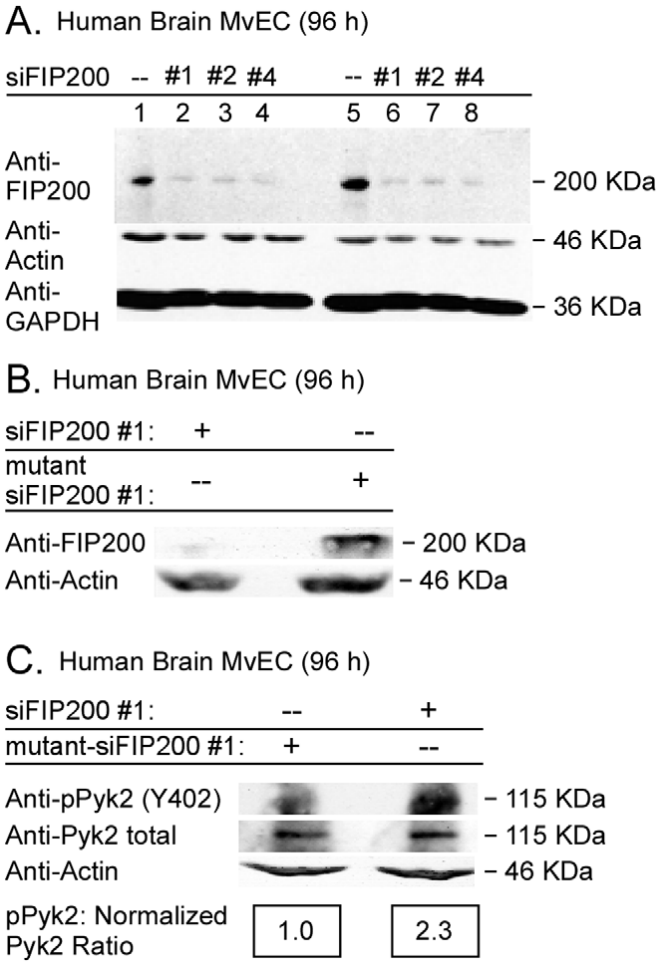
Downregulation of FIP200 protein with siRNA enhances Pyk2 activity without affecting FAK activity in primary human brain MvEC. A–C, Primary human brain MvEC were plated in complete media (10% FSB) and treated with siFIP200 #1, #2, or #4, or with control liposomes or mutant siFIP200 #1 for 96 h, detergent lysed, the lysate resolved on disulfide-reduced 7.5% SDS-PAGE and blotted with the indicated antibodies. In Panel C, the densitometric ratio of pPyk2/normalized to Pyk2 was set at 1.0 for the cells treated with mutant siFIP200, and the ratio for the cells treated with specific siFIP200 expressed relative to the ratio for the cells treated with mutant siFIP200. The experiment was repeated and representative blots are shown.

Somewhat surprisingly, we found that FIP200 downregulation in U-87MG cells, E6/E7 immortalized astrocytes, and primary human brain MvEC propagated in complete media did not affect either the levels of FAK activity (as determined by the level of autophosphorylation at Y397) or the levels of FAK protein (Figures 4A & 4B). In contrast, downregulation of FIP200 enhanced the activity of Pyk2 in all three cell types although it did not enhance the levels of Pyk2 protein (Figures 4C and 5). The levels of autophosphorylation on Y402 relative to normalized Pyk2 protein were approximately 2-fold higher in the U-87MG cells and the primary human brain MvEC, and 4-fold higher in the E6/E7 cells at 48h after knockdown of FIP200. At the same time-point, the levels of autophosphorylation were approximately 4-fold higher in U-87MG cells propagated in suspension (data not shown).

**Figure 6:**
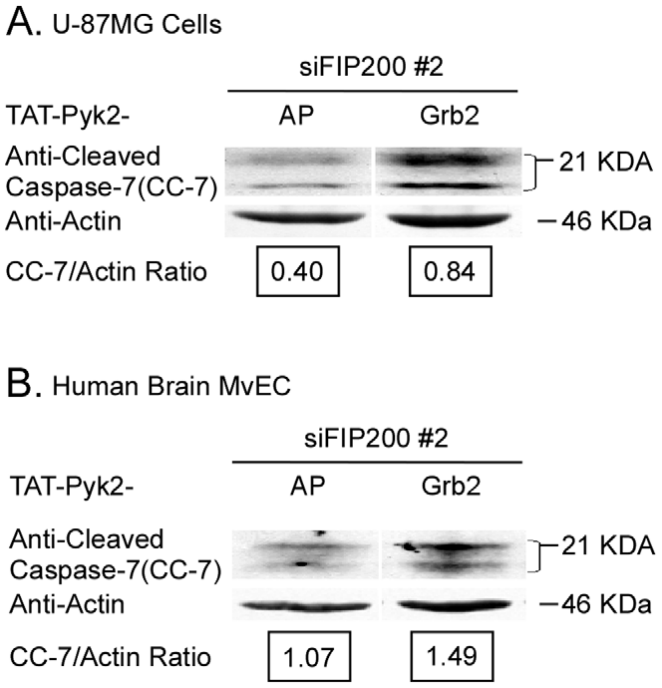
A TAT-Pyk2 fusion protein containing the autophosphorylation site inhibits the pro-apoptotic effect observed with the downregulation of FIP200-protein. (A) U-87MG glioblastoma cells plated in complete media (10% FBS) (20 h) were treated with the indicated siRNA for 48 h, the media removed and the cell monolayer treated with 150 nM TAT-Pyk2 fusion protein containing the autophosphorylation site (AP) or the potential Grb2 binding site (Grb2) for 1 h, followed by fresh media and siRNA for 38 h, detergent lysis, and immunoblotting with the indicated antibodies. B, Human brain MvEC (HBMvEC) were plated in complete media (20 h), treated with the indicated siRNA (50 h), the media removed and the cell monolayer treated with 75 nM TAT-Pyk2-fusion protein (1 h), followed by fresh media and siRNA for 36 h, detergent lysis and immunoblotting with the indicated antibodies. The experiment was repeated and representative blots are shown.

As the Pyk2 autophosphorylation site has been shown to be necessary for the pro-apoptotic effect of Pyk2 [11], [18], we investigated whether a TAT-Pyk2 fusion protein containing the autophosphorylation site (abbreviated TAT-Pyk2-AP) [31] could block the pro-apoptotic effect of downregulation of FIP200. A TAT-Pyk2 fusion protein containing the Grb2 binding site (Y881) was used as a control [31]. The TAT-Pyk2-AP fusion protein (150 nM) significantly reduced the pro-apoptotic effect of downregulation of FIP200 in both the U-87MG glioblastoma cells (Figure 6A) and the primary human brain MvEC (Figure 6B). These data suggest that the apoptosis associated with the downregulation of FIP200 protein in the U-87MG cells and primary brain MvECs propagated in complete media most likely requires activation of Pyk2.

FIP200 also has been shown to promote the activity of S6 kinase by associating with and inhibiting the function of the TSC1&2 complex of proteins [32]. To determine the effects of FIP200 downregulation on S6 kinase activity, the cell lysates were blotted for phospho-p70 S6 kinase (Thr389). We found a small reduction (≈30% based on densitometry when normalized to the loading control cortactin) in S6 kinase activity on FIP200 downregulation in the U-87MG cells at 96 h (data not shown), suggesting that a reduction in S6 kinase activity may contribute in part to the decreased proliferation we observed on FIP200 downregulation.

### Greater Amounts of FIP200 Protein are Co-Immunoprecipitated with Pyk2 than with FAK

**Figure 7:**
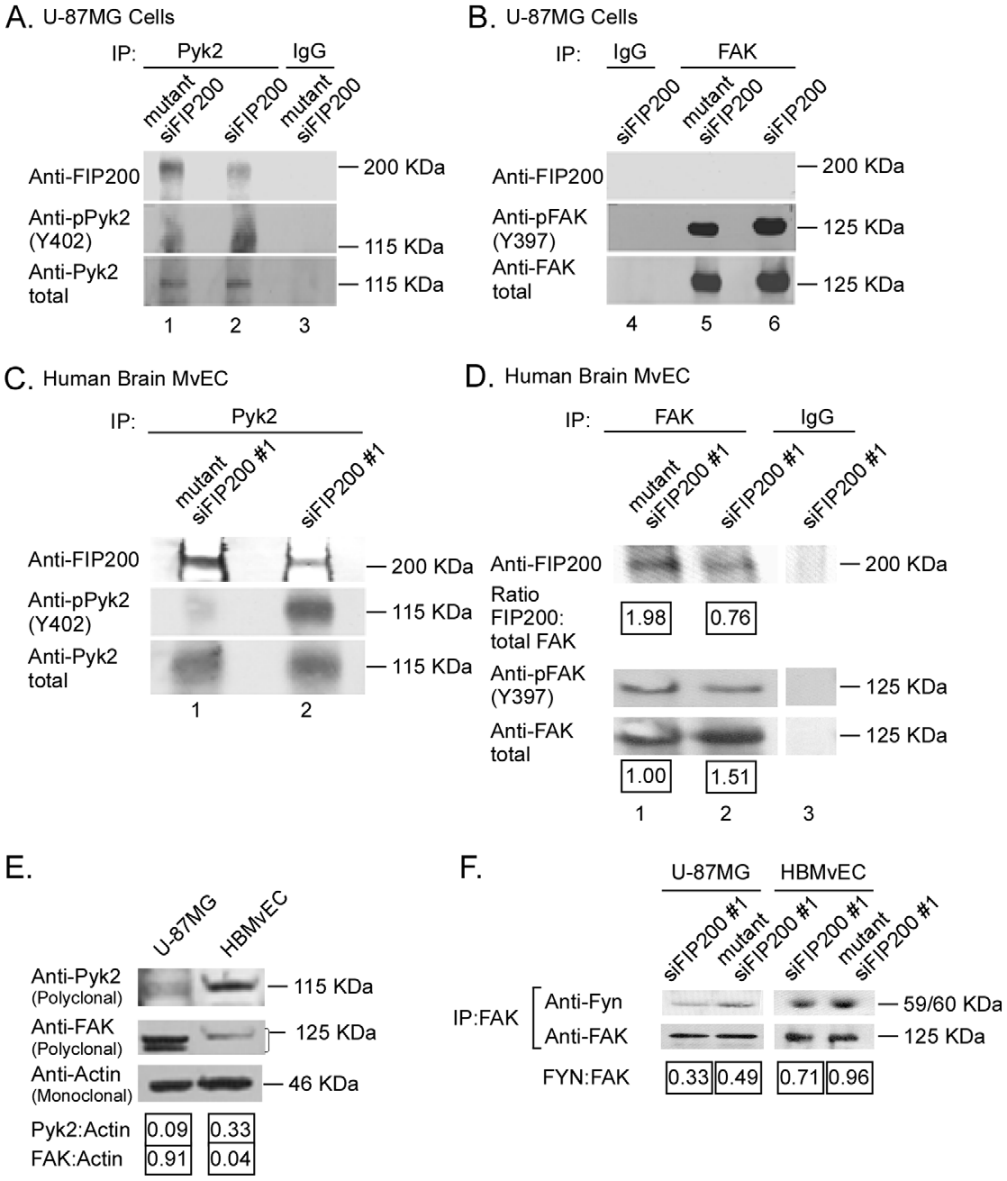
FIP200 protein predominantly associates with Pyk2 in the glioblastoma cells and primary human brain MvEC. A–D, U-87MG glioblastoma cells (A&B) and primary human brain MvEC (C&D) were plated in complete media for 20 h, treated with siFIP200 or mutant siFIP200 for 96 h, detergent lysed, and immunoprecipitated with the indicated antibody. The immunoprecipitates were subjected to 7.5% disulfide-reduced SDS-PAGE and immunoblotted with the indicated antibodies, as described in the Materials and Methods. Panels A & B are from the same gel. (E) U-87MG cells and human brain MvEC plated in complete media were detergent lysed, subjected to 8% SDS PAGE, and immunoblotted with the indicated antibodies. (F) U-87MG cells and human brain MvEC plated as above and treated with the indicated siRNA for 72 h were detergent lysed, immunoprecipitated with anti-FAK antibody, the immunoprecipitate subjected to SDS-PAGE, followed by immunoblotting with the indicated antibodies. The experiment was repeated 2× and representative blots are shown.

Immunoprecipitation of Pyk2 from lysates of U-87MG cells, resulted in co-immunoprecipitation of FIP200 (Figure 7A, lane 1) and downregulation of FIP200 by treatment of the cells with siFIP200 reduced the 200-kDa band detected in the Pyk2 immunoprecipitate by an estimated 70% (based on densitometry) (Figure 7A, lane 2). Immunoprecipitation of FAK from lysates of U-87MG cells did not result in detectable coprecipitation of FIP200 (Figure 7B) even after prolonged exposure of the autoradiographs (data not shown). Thus, there is an apparent preferential association of FIP200 with Pyk2 in these cells.

In contrast, FIP200 co-immunoprecipitated with both Pyk2 and FAK from lysates of the primary human brain MvEC (Figure 7C, lane 1, and Figure 7D, lane 1, respectively), although lesser amounts of FIP200 were associated with FAK than Pyk2. Treatment of the cells with siFIP200 reduced the 200-kDa band associated with Pyk2 (Figure 7C, lane 2) and with FAK (Figure 7D, lane 2) by an estimated 70% and 60% respectively (based on densitometric analysis). The FIP200 downregulation increased Pyk2 activity (pPyk2(Y402) blot) (Figure 7C, lane 2), but not FAK activity (pFAK(Y397) blot) (Figure 7D, lane 2) in the MvEC used in this assay. Thus, there also is an apparent preferential association of FIP200 with Pyk2 in the MvEC. The levels of total Pyk2 protein and total FAK protein in the U-87MG and MvEC cell lysates are shown in Figure 7E.

### A Reduction in the Association of Fyn with FAK Occurs on FIP200 Downregulation

Activated Pyk2 may compete with FAK for binding partners and thereby decrease the FAK-promoted pro-survival signal in cells [11], [13]. It has been demonstrated that Pyk2 activation is associated with a reduction in the amounts of cellular Src or Fyn associated with FAK in other cell types [11], [13]. As we have shown previously that Fyn, Lyn and c-Src are the predominant Src family members that are expressed in glioblastoma tumors and in the U-87MG cell line [33], [34], we examined the effects of FIP200 downregulation on the association of these molecules with FAK. We found that FIP200 downregulation reduced the amount of Fyn associated with FAK in the U-87MG cells (60% decrease by normalized densitometry) (Figure 7F), but did not reduce the association of Lyn or c-Src with FAK (data not shown). Similarly, in the human brain MvEC, we found a reduction in the amount of Fyn (25% decrease) associated with FAK on FIP200 downregulation, and no decrease in Lyn or c-Src association with FAK (data not shown). Thus, one possible explanation for the observed effects on FIP200 downregulation is that pPyk2 is competing with FAK for Src family binding partners, but we have not ruled out other possible explanations.

### FIP200 and Pyk2 Localization is Largely Cytoplasmic and Diffuse

On analysis of the cellular distribution of FIP200 using double-label immunofluorescence, we found that the expression was largely cytoplasmic and diffuse in the U-87MG cells and primary human brain MvEC (Figures S3A & S3C, arrows) as reported previously in other cell types [13]. We did not observe localization of FIP200 to focal adhesions. Similar to FIP200, we found that total Pyk2 localization was cytoplasmic and diffuse (Figures S3A–D), independent of FIP200 downregulation and consistent with that reported previously in fibroblasts and PC12 cells [13], [35]. FIP200 downregulation did not appear to affect the localization of Pyk2. As expected, FAK protein largely co-localized with vinculin to focal adhesions in both cell types, independent of FIP200 downregulation (data not shown).

**Figure 8:**
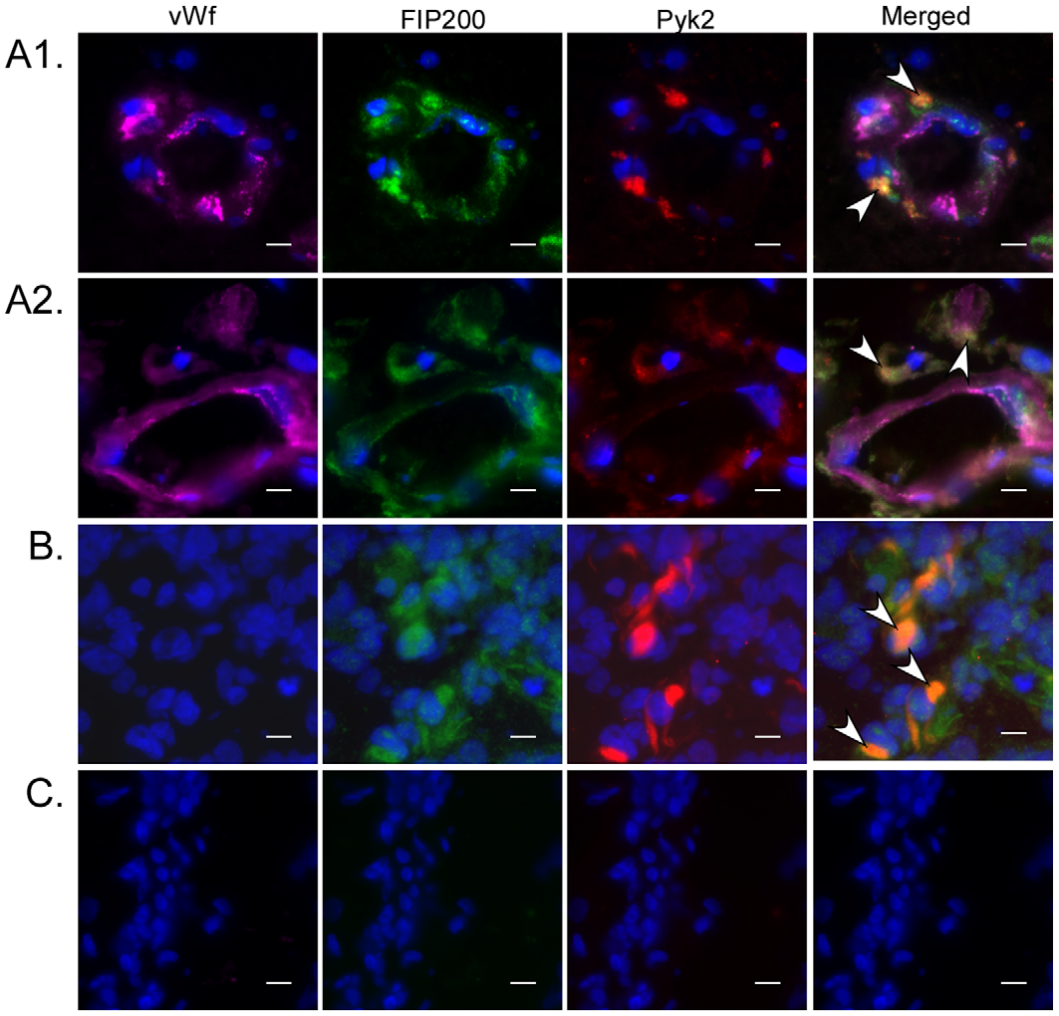
Co-Expression of FIP200 and Pyk2 in the cytoplasm of tumor cells and of tumor-associated endothelial cells. Frozen sections of four glioblastoma tumors were reacted with antibodies directed toward vWf (pink), FIP200 (green) and Pyk2 (red), followed by reaction with Alexa 633-, Alexa 563- and Alexa 488-conjugated secondary antibodies, and DAPI staining, as described in the Materials and Methods. Images were viewed and photographed on a Leica DMB microscope at 400× magnification. A1 & A2, triple-labeled tumor-associated endothelial cells; B, double-labeled tumor cells; and C, secondary antibodies alone followed by DAPI staining. Merged images are shown at the reader's far right.

Analysis of the cellular localization of these proteins in four frozen glioblastoma biopsy samples by triple-label immunofluorescence indicated cytoplasmic co-expression of FIP200 and Pyk2 in endothelial cells in tumor-associated blood vessels identified by vWf expression (Figure 8A1&2), suggesting that FIP200 and Pyk2 are localized in a manner that is consistent with their potential association. In the tumor cell compartment of all samples, we found cytoplasmic co-expression of FIP200 and Pyk2 in some tumor cells (Figure 8B), also suggesting that FIP200 and Pyk2 are localized in a manner that is consistent with potential association. Nuclear localization of FIP200 also was observed in some tumor cells (data not shown). The cytoplasmic localization of Pyk2 in the tumor-associated endothelial cells and in the tumor cells in frozen glioblastoma biopsies is consistent with the report of another group that examined Pyk2 expression in glioblastoma biopsies [36].

## Discussion

Our demonstration that specific downregulation of endogenous FIP200 protein can increase Pyk2 activity and induce apoptosis adds to the body of evidence that FIP200 can regulate Pyk2 and underscores the importance of FIP200 in the regulation of cell survival. Collectively, our results indicate that FIP200 regulation of Pyk2 can play an important role in the survival of glioblastoma tumor cells and suggests the intriguing possibility that this mechanism may contribute to sustained tumor growth by promoting not only the survival of tumor cells but also the intra-tumor glial cells and the tumor-associated endothelial cells.

These results underscore the importance of FIP200 in the regulation of cell survival in that they demonstrate that downregulation of FIP200 can induce apoptosis. The potential role of pPyk2 in promoting the apoptosis induced by FIP200 downregulation was indicated by the ability of a TAT-Pyk2 fusion protein containing the autophosphorylation site to inhibit the pro-apoptotic effect, whereas a TAT-Pyk2 fusion protein containing the potential Grb2 binding site (Y881) did not. This is consistent with the prior report that the N-terminus and the kinase domains of Pyk2, but not the C-terminus, are necessary for the pro-apoptotic effect of Pyk2 [11]. Several other mechanisms have been implicated in FIP200 regulation of apoptosis in other cell types. In the FIP200-null mouse embryos, no changes in Pyk2 activity were detected in the hepatocytes or cardiomyocytes and the enhanced apoptosis was attributed to a decrease in S6 kinase activity [5]. The TSC1&2 complex is thought to negatively regulate cell proliferation through S6 kinase, mTOR, and 4E-BP1 [5], [32]. On analysis of the p70 S6 kinase activity in the glioblastoma cells we did find that there was a slight reduction (an estimated 30%) in this activity on downregulation of FIP200. This small decrease in S6 kinase activity could contribute to the inhibition of proliferation we observed on FIP200 downregulation but the current results suggest that the mechanism by which FIP200 regulates apoptosis in glioblastoma tumor cells and brain MvECs differs from that in the embryonic murine cardiocytes and hepatocytes in that it requires Pyk2 activity. It also has been reported that FIP200 upregulates the expression of the retinoblastoma (*Rb*) gene [26], [37], thus the downregulation of endogenous FIP200 protein in our cells could potentially inhibit cell proliferation by decreasing the *Rb* gene expression. Although the association of FAK with the death domain kinase receptor interacting protein (RIP) is thought to prevent the pro-apoptotic signal of RIP [38], we could not detect an association of FAK with RIP in the U-87MG cells by co-immunoprecipitation (D. Wang and C.L. Gladson unpublished observation).

Although both Pyk2 and FAK were expressed in the glioblastoma cells and human brain MvEC, we found that FIP200 preferentially co-immunoprecipitated with Pyk2 in the absence of FIP200 downregulation in the unmanipulated U-87MG glioblastoma and brain MvEC cells propagated in complete media. The association of the endogenous FIP200 with Pyk2 in the lysates of the unmanipulated glioblastoma cells and human brain MvEC propagated in complete media suggests that the FIP200 regulation of Pyk2 activity in these cells may be a direct effect. The interaction of FIP200 with Pyk2 has been shown to inhibit Pyk2 activity in other cell types [2] and Pyk2 activation has been shown to promote its dissociation from FIP200. Thus, it is possible that downregulation of the endogenous FIP200 may lead to exposure of the autophosphorylation site permitting autophosphorylation of Pyk2 [2], [13], [39].

The activities of Pyk2 and FAK and their contribution to specific cell functions, including apoptosis, are regulated in large part by their shuttling between different compartments of the cell. The largely cytoplasmic and diffuse pattern of FIP200 that we observed on immunofluorescence analysis of both the U-87MG cells and the primary human brain MvECs is consistent with the pattern reported for fibroblasts [2], [13]. The distribution of Pyk2 also was predominantly cytoplasmic and diffuse, which is consistent with the preferential co-precipitation of FIP200 with Pyk2 we observed, whereas FAK was largely localized to the focal adhesions and FIP200 was not detectable at these sites. The regulation of localization of FAK and Pyk2 to focal adhesions can be independently influenced by the presence or absence of specific binding partners, for example, gelsolin has been reported to bind Pyk2 but not FAK [40] as well as differences in the affinity of FAK and Pyk2 for shared binding partners within the focal adhesions, such as paxillin [41]–[43]. FIP200 localization to the nucleus has been reported in other cell types [26], emphasizing a potential role for FIP200 in regulation of transcription through, for example, its potential interaction with Rb, which is a regulator of transcription. We did not observe a clear-cut association of FIP200 with the nuclei of the U-87MG or primary human MvECs, but we did not utilize biochemical assays to explore this possibility and cannot rule out a partial nuclear localization. However, the pattern of expression of FIP200 in the tumor cells in the glioblastoma biopsies was heterogeneous. Although the pattern was predominantly cytoplasmic, nuclear staining was observed and partial FIP200 localization to the nucleus was observed in tumor cells in 10 of 36 biopsy samples. In contrast, in the tumor-associated endothelial cells of glioblastoma biopsies, the distribution of FIP200 was largely cytoplasmic with only rare nuclear staining.

We observed a reduced association of Fyn with FAK on FIP200 downregulation in both U-87MG cells and the brain MvEC although the levels of activity of FAK and its expression were unaffected by the downregulation. This supports the concept that activated Pyk2 competes with FAK for binding partners that affect downstream signaling pathways, as shown in other cell types by Zhao and colleagues [13]. Activated Pyk2 can signal the activation of p38MAP kinase [20], a MAP kinase that can promote apoptosis [44], and Pyk2 induction of apoptosis in cardiomyocytes requires Src activation of p38MAP kinase [18]. FAK can promote cell proliferation and cell survival through the activation of other MAP kinase(s) in a cell-type and experimental context-dependent manner reviewed in [3], [45].

It should be emphasized that there was heterogeneity in the levels of expression of FIP200 in the tumor cells and that FIP200 was not detectable in all of the tumor cells within a single biopsy specimen. In addition, we did not detect FIP200 protein in tumor cells in 8 of 36 of the glioblastoma biopsies, although FIP200 was detected in scattered glial cells and the endothelial cells in the tumor-associated vessels in 3 of these 8 tumors. Currently, it is not known whether the failure to detect FIP200 protein in tumor cells in eight of the glioblastoma biopsy samples is due to a gene deletion in these cells or an environmentally triggered reduction in the levels of FIP200 mRNA or protein.

The location of the *FIP200* gene on chromosome 8q11 [6], [8], [9] has led other investigators to formulate the hypothesis that FIP200 could be an “anti-tumor” or “tumor suppressor” gene. Recently, however, Wei and colleagues [23] created a conditional knockout of FIP200 in the MMTV-Cre model of breast cancer and showed no promotion of tumorigenesis, suggesting that FIP200 inactivation alone does not promote tumor formation. In addition, a prior report demonstrated that overexpression of FIP200 in breast cancer cells resulted in an inhibition of both cell cycle progression and clonogenic cell survival due to FIP200 promotion of p21 expression [7]. It is possible that the level of FIP200 protein in cells, and the cell type, influences its function. For example, the kinetics of FIP200 association with some of its binding partners, such as Pyk2, FAK or the p21 or Rb promoter, could be altered when FIP200 is overexpressed and this could be affected by the experimental context and the specific cell-type. Taken together with the expression of FIP200 in the tumor-associated endothelial cells in the glioblastoma tumors but not the normal brain, these data underscore the importance of elucidation of the factors that regulate the expression of FIP200.

## Materials and Methods

### Reagents

The following antibodies were purchased, rabbit anti-FAK IgG (Upstate Biotechnology, Cat# 06-543); mouse mAb anti-FAK (Santa Cruz Biotechnology; clone # H-1); rabbit anti-phospho-FAK (pY397) (Chemicon, Inc.); mouse mAb anti-Pyk2 (BD Biosciences-Transduction Laboratories; clone 11); rabbit anti Pyk2 (Santa Cruz, #SC9019), and rabbit anti-phospho-Pyk2 (pY402) (BioSource International and Upstate Biotechnology). Two rabbit anti-FIP200 antibodies were used. One was purchased from (ProteinTech, Chicago, IL) and the other, a rabbit anti-FIP200 antibody directed toward the C-terminus, has been described previously [2]. Mouse mAb anti-death domain kinase receptor interacting protein (RIP) (BD Transduction Laboratories); rabbit anti-cleaved caspases 7 (Calbiochem); mAb anti-actin (Sigma), rabbit monoclonal anti-phospho-p70 S6 kinase (Thr389) (Upstate Biotechnology), mAb anti-glyceraldehyde-3-phosphate-dehydrogenase (GAPDH) (Fitzgerald Laboratories), mouse anti-α-tubulin (GeneTex, DM1A) and rabbit antibodies toward Fyn, Lyn and c-Src (Santa Cruz Biotechnology) also were purchased. Control normal rabbit IgG and normal IgG were purchased from Santa Cruz Biotechnology.

### Cell culture

U-87MG human glioblastoma cells were obtained from the ATCC and propagated as described [33]. Human astrocytes immortalized with the telomerase gene and the E6 and E7 viral genes have been characterized previously and were propagated as described [28], and primary human astrocytes were purchased from Cell Systems (Kirkland, WA) and propagated as recommended. Highly similar results were found on FIP200 downregulation in both the immortalized and the primary human astrocytes. SNB19 human glioblastoma cells were cultured as described previously [46], and were a kind gift from Dr. Jasti Rao (University of Illinois, Peoria, IL). The 827 and 905 human glioblastoma stem cells were cultured as described previously [47]. Primary human brain MvEC were obtained from Cell Systems and propagated as directed [29], [30].

### Human tissues

Frozen glioblastoma and anaplastic astrocytoma tumor tissue, paraffin sections of glioblastoma tumor, and normal brain tissue (tissue adjacent to seizure foci resected to gain access to the seizure foci), were obtained from surgical samples with the approval of the Institutional Review Board of the University of Alabama at Birmingham (IRB number: X050415007) or the Cleveland Clinic (IRB number: 2559). All tissue banked at the University of Alabama at Birmingham and the Cleveland Clinic was with the written consent from the patient. De-identified tissue was provided by the tissue bank at the University of Alabama at Birmingham or the Cleveland Clinic. Glioblastoma tumor (Grade IV of IV) and anaplastic astrocytoma tumor (Grade III of IV) were diagnosed according to the most recent World Health Organization Classification of Tumors of the Central Nervous System [48]. Frozen tumor and normal brain tissues were lysed in RIPA lysis buffer with protease inhibitors as described [34].

### Immunohistochemistry

Paraffin and frozen sections (7 microns thick) were stained as described previously [34] with the Avidin/Biotin Kit (Vector Laboratories) as recommended. Rabbit anti-FIP200 was utilized at 0.2 µg/ml for paraffin sections and 1.13 µg/ml for frozen sections as described previously [10], and the following antibodies mAb anti-Pyk2, rabbit anti-vWf, goat anti-vWf, and the negative control IgG were utilized at a 1∶100 dilution of the stock. Sections were viewed and photographed using a Leica DM4000B microscope.

### Downregulation of FIP200 with siRNA

Three validated duplex siRNAs for FIP200 were purchased from Qiagen (Valencia, CA): siFIP200 #1 (Cat. # SI02664571), target sequence CTGGGACGCATACAAATCCAA; siFIP200#2 (Cat #SI02664578), target sequence ACGCAAATCAGTTGATGATTA; and siFIP200 #4 (Cat #SI00108122). To further substantiate specific downregulation of FIP200, siFIP200 #1 and #2 duplex oligonucleotides with one or two base pair mutations (mutant siFIP200) were purchased from Qiagen and used as controls. The target of the mutated siFIP200#1 is CTGGGACGGATACAAATCCAA; and the target of mutated siFIP200#2, ACGCAAGTCCGTTGATGATTA. Control non-silencing siRNA also was purchased from Qiagen. Transient transfection of siRNA was carried out using the HiPerfect transfection reagent (Qiagen), according to the manufacturer's instructions, with empty liposomes being used as a negative control. The efficiency of the downregulation of FIP200 in the different cell types was confirmed and monitored by western blot analysis. No effects on the morphology of the cells were observed.

### Immunoblot analysis

Immunoblotting was carried out as described previously [17]. The cells were lysed in the Cell Lytic-M reagent lysis buffer (Sigma) or with 1% NP40 buffer with protease inhibitors [17]. Immunoblotting after lysis of the cells in RIPA lysis buffer yielded similar results except that the blots were not as clean. The rabbit anti-FIP200 antibody directed toward the C-terminus [2] was used at a 1∶1000 dilution and the commercial anti-FIP200 at 0.2 microgram/ml. Band intensities were measured by densitometry and normalized to the intensity of the loading control, *i.e.*, antibody directed to actin, α-tubulin or cortactin. FAK activity was detected by blotting using an antibody specific for pFAK(Y397), and then stripping and reprobing for total FAK and a loading control. Pyk2 activity was detected by blotting using an antibody specific for pPyk2(Y402), then stripping and reprobing for total Pyk2 and a loading control. The activity of FAK and Pyk2 was determined by normalizing the intensity of the pFAK(Y397) and pPyk2(Y402) bands to total FAK or Pyk2 bands, respectively.

### Cell proliferation and apoptosis assays

Immortalized astrocytes, U-87MG cells, and human brain MvEC were plated on 12- or 24-well plates in complete media (10% FBS) and then treated with siRNA for the indicated times; the medium was changed and fresh siRNA added every three or four days. To assess proliferation, adherent cells were harvested with trypsin/IEDTA, stained with trypan blue and viable cells counted using a hematocytometer. The TUNEL apoptosis assay was performed as described previously [29], [30].

### Immunoprecipitation

U-87MG cells or human brain MvEC were plated in complete media at 80% confluence for 20 h, followed by siRNA treatment for the indicated number of days. The cells were then lysed using 1% NP40 Lysis Buffer with protease inhibitors as described [33]. For immunoprecipitation analysis equivalent amount of protein lysate (50 to 200 µg) from each sample was incubated with 5–10 µg of the appropriate antibody followed by pull-down using TrueBlot anti-rabbit IP beads (eBiosciences) overnight at 4°C. The immunoprecipitates were washed 3 times with PTO buffer (PBS with 0.1% ovalbumin and 0.5% Tween 20) and 3 times with PT buffer (PBS with 0.5% Tween 20) and subjected to 7.5% disulfide-reduced SDS-PAGE, transferred to PVDF membrane and immunoblotted as described [33].

### Immunofluorescence analysis

Cells were plated in complete media (10% FBS) and treated with siRNA for the indicated times. Frozen tissue was sectioned at 7 microns. Immunofluorescence was performed as described previously [49]. A Leica DMF microscope was used for viewing and photomicrographs were obtained using a 40× oil objective.

### TAT-Pyk2 Fusion Proteins

The following constructs in the pTatHA bacterial expression vector were provided by Dr. Carl Nathan (Weil Medical College of Cornell University, NY): nt 1102–1557, coding for amino acids 365–518 and containing the autophosphorylation site (abbreviated AP), nt 1741–2099, coding for amino acids 581–700 and containing the putative phosphatidyl inositol 3′ phosphate (PI3) kinase binding domain (abbreviated PBM); and nt 2617–2986, coding for amino acids 873–995 and containing the Grb2 binding site (abbreviated Grb2BS) [31]. The constructs were grown in BL21 DE3 *E. coli*, and the recombinant proteins purified as described [31]. TAT fusion proteins were used for these studies as they have been shown to transduce nearly 100% of mammalian cells regardless of cell type [50], [51]. Both the TAT-Pyk2 fusion proteins are well characterized and do not exhibit toxicity in various *in vitro* and *in vivo* models [31], [51].

## Supporting Information

Figure S1 **FIP200 expression in glioblastoma tumor and normal brain biopsies.** Tissues were detergent lysed and 200 micrograms of each sample electrophoresed on an 6% disulfide-reduced SDS-PAGE, transferred to Immobilon membrane, and then immunoblotted with the indicated antibodies, as described in the Materials and Methods. The densitometirc reading for the FIP200 band in each sample was normalized to the actin band that served as a loading control.
(TIF)

Figure S2 **FIP200 expression in glioblastoma cells and primary brain microvessel endothelial cells (MvEC).** U-87MG and SNB19 human glioblastoma cells plated as a monolayer in complete media (A), 905 and 827 human glioblastoma stem cells propagated as neurospheres in serum-free media (A), as well as primary human brain MvEC plated as a monolayer in complete media (B), were detergent lysed, equivalent amount of lysate subjected to 7.5% SDS-PAGE, and the gels immunoblotted with the indicated antibodies as described in the Materials and Methods. The experiment was repeated and representative blots are shown.
(TIF)

Figure S3 **FIP200 and Pyk2 are localized in a cytoplasmic, diffuse manner in glioblastoma cells and human brain MvEC.** U-87MG glioblastoma cells or primary human brain MvEC were plated onto chamberslides in complete media (10% FBS) or in complete media with 10 ng/ml VEGF, and 5 ng/ml bFGF, respectively, for 20 h, treated with siFIP200 or mutant siFIP200 (labeled control siRNA) for 48 h, fixed, and subjected to double-label immunofluorescence, as described in the Materials and Methods. MAb anti-vinculin was detected with Alexa 488-conjugated anti-mouse antibody (green fluorescence), and rabbit anti-FIP200, and anti-total Pyk2 antibodies were detected with anti-rabbit Alexa 594-conjugated antibody (red fluorescence). Merged images are shown. Arrows denote a diffuse cytoplasmic localization (FIP200 and Pyk2), and arrowheads denote a localization at focal adhesions (vinculin). Cells were viewed and photographed using a Leica DMR microscope. All panels, magnification 400×. The experiment was repeated and representative photomicrographs are shown.
(TIF)

Table S1 **FIP200 protein expression in cells treated with siFIP200.** The cells were propagated in complete media and treated with siFIP200 #1 or #2 at 20 nM, empty liposomes or mutant siFIP200 as described in the Materials and Methods section for the indicated time periods. The cells were then harvested and FIP200 protein expression evaluated by western blotting. Densitometric readings of the FIP200 band on the blots were normalized to the loading control protein (actin, GAPDH or cortactin). The normalized FIP200 protein expression in the siFIP200-treated cells is shown as a percentage of the normalized FIP200 expression in the control cells treated with *liposome or ^+^mutant siFIP200. Abbreviations: HBMvEC, primary human brain microvessel endothelial cells.
(TIF)
