# Supplementary figures and images for: Downregulation of FIP200 Induces Apoptosis of Glioblastoma Cells and Microvascular Endothelial Cells by Enhancing Pyk2 Activity
Source: PLoS One. 2011 May 13;6(5):e19629. doi: 10.1371/journal.pone.0019629 (PMC3094350; doi:10.1371/journal.pone.0019629)

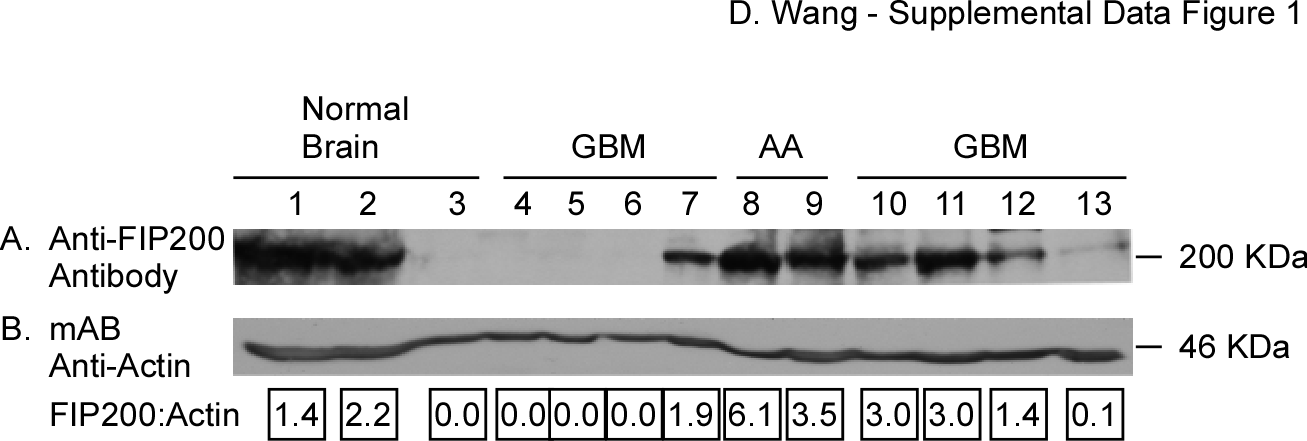

Supplement: Figure S1 — FIP200 expression in glioblastoma tumor and normal brain biopsies. Tissues were detergent lysed and 200 micrograms of each sample electrophoresed on an 6% disulfide-reduced SDS-PAGE, transferred to Immobilon membrane, and then immunoblotted with the indicated antibodies, as described in the Materials and Methods. The densitometirc reading for the FIP200 band in each sample was normalized to the actin band that served as a loading control. (TIF) [file pone.0019629.s001.tif]

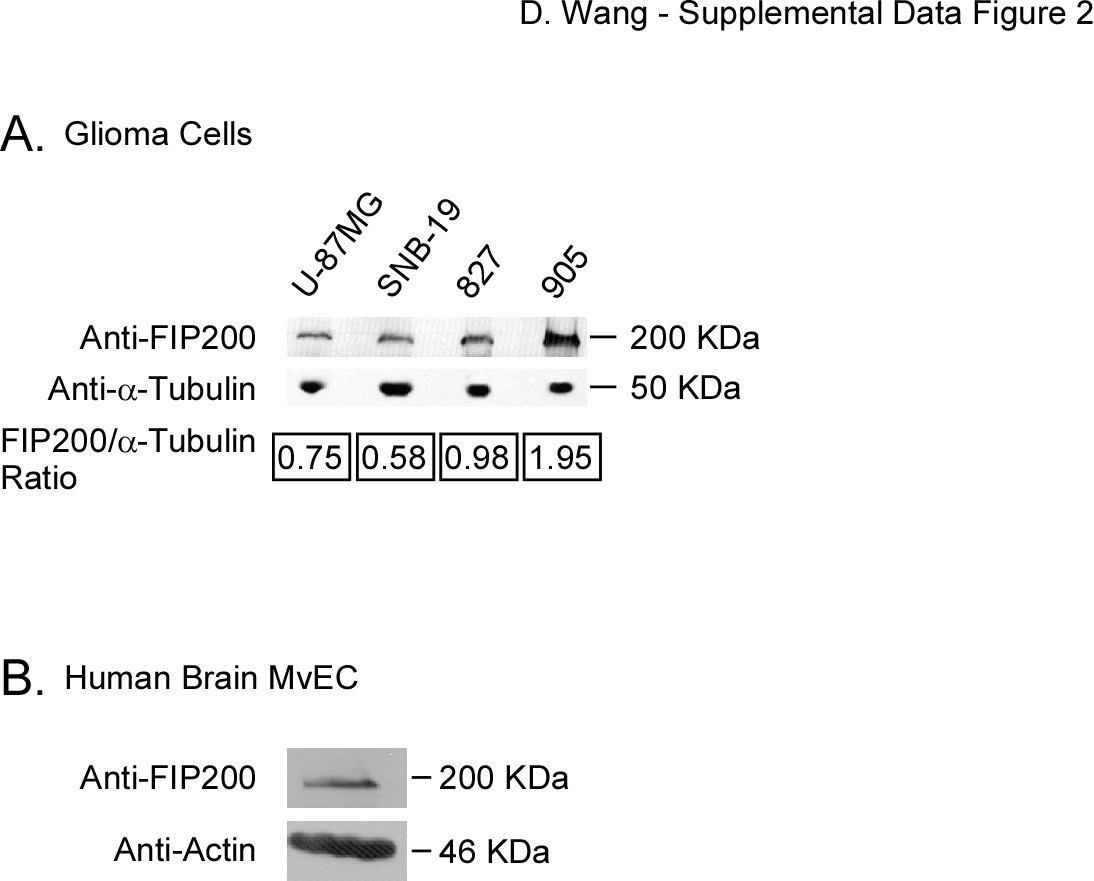

Supplement: Figure S2 — FIP200 expression in glioblastoma cells and primary brain microvessel endothelial cells (MvEC). U-87MG and SNB19 human glioblastoma cells plated as a monolayer in complete media (A), 905 and 827 human glioblastoma stem cells propagated as neurospheres in serum-free media (A), as well as primary human brain MvEC plated as a monolayer in complete media (B), were detergent lysed, equivalent amount of lysate subjected to 7.5% SDS-PAGE, and the gels immunoblotted with the indicated antibodies as described in the Materials and Methods. The experiment was repeated and representative blots are shown. (TIF) [file pone.0019629.s002.tif]

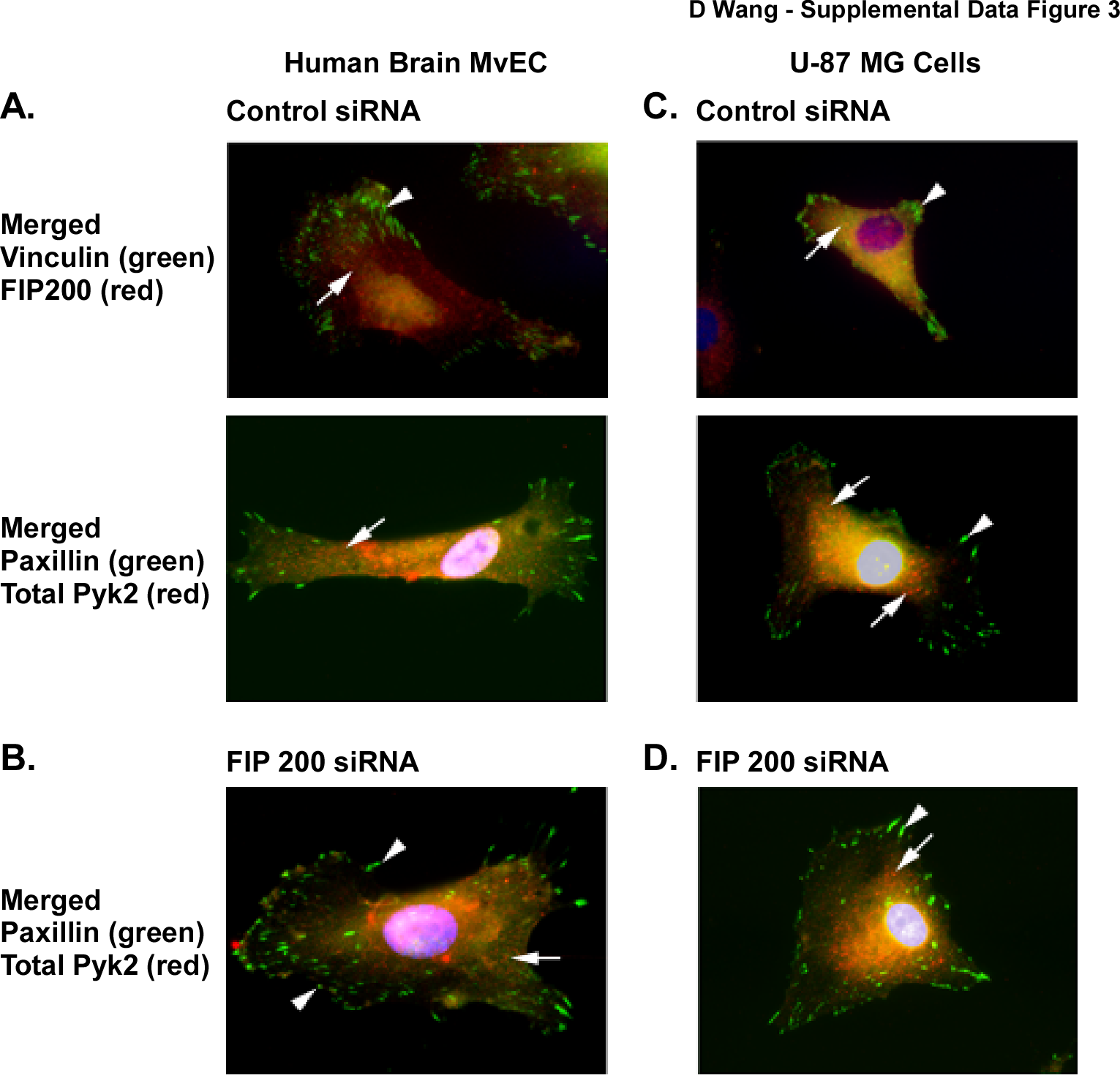

Supplement: Figure S3 — FIP200 and Pyk2 are localized in a cytoplasmic, diffuse manner in glioblastoma cells and human brain MvEC. U-87MG glioblastoma cells or primary human brain MvEC were plated onto chamberslides in complete media (10% FBS) or in complete media with 10 ng/ml VEGF, and 5 ng/ml bFGF, respectively, for 20 h, treated with siFIP200 or mutant siFIP200 (labeled control siRNA) for 48 h, fixed, and subjected to double-label immunofluorescence, as described in the Materials and Methods. MAb anti-vinculin was detected with Alexa 488-conjugated anti-mouse antibody (green fluorescence), and rabbit anti-FIP200, and anti-total Pyk2 antibodies were detected with anti-rabbit Alexa 594-conjugated antibody (red fluorescence). Merged images are shown. Arrows denote a diffuse cytoplasmic localization (FIP200 and Pyk2), and arrowheads denote a localization at focal adhesions (vinculin). Cells were viewed and photographed using a Leica DMR microscope. All panels, magnification 400×. The experiment was repeated and representative photomicrographs are shown. (TIF) [file pone.0019629.s003.tif]

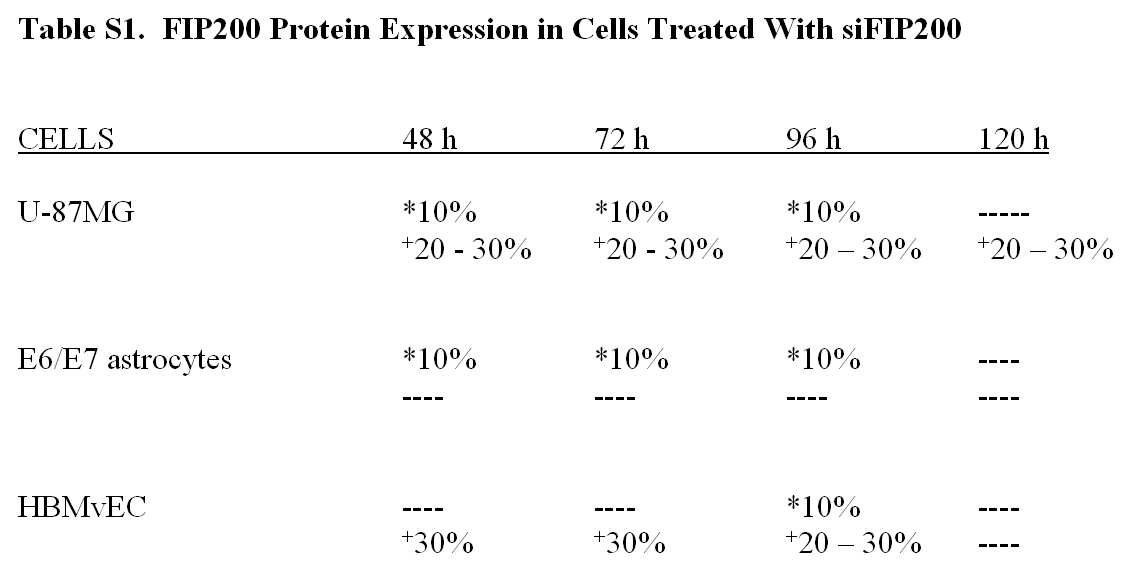

Supplement: Table S1 — FIP200 protein expression in cells treated with siFIP200. The cells were propagated in complete media and treated with siFIP200 #1 or #2 at 20 nM, empty liposomes or mutant siFIP200 as described in the Materials and Methods section for the indicated time periods. The cells were then harvested and FIP200 protein expression evaluated by western blotting. Densitometric readings of the FIP200 band on the blots were normalized to the loading control protein (actin, GAPDH or cortactin). The normalized FIP200 protein expression in the siFIP200-treated cells is shown as a percentage of the normalized FIP200 expression in the control cells treated with *liposome or +mutant siFIP200. Abbreviations: HBMvEC, primary human brain microvessel endothelial cells. (TIF) [file pone.0019629.s004.tif]
